# Skeletal-Related Events in Renal Cell Carcinoma: Prediction With Alkaline Phosphatase (ALP), C-reactive Protein (CRP), Haemoglobin (Hb) and Erythrocyte Sedimentation Rate (ESR) (A.C.H.E.) Score for Risk Stratification

**DOI:** 10.7759/cureus.40835

**Published:** 2023-06-22

**Authors:** Yashasvi Singh, Sasanka Kumar Barua, Sameer Trivedi, Rajeev TP, Manash Pratim Kashyap, Lalit Kumar Agrawal, Ujjwal Kumar Pathak, Neha Garg

**Affiliations:** 1 Department of Urology, Institute of Medical Sciences, Banaras Hindu University, Varanasi, IND; 2 Department of Urology, Gauhati Medical College and Hospital, Guwahati, IND; 3 Department of Statistics, Assam Down Town University, Guwahati, IND; 4 Department of Medicinal Chemistry, Institute of Medical Sciences, Banaras Hindu University, Varanasi, IND

**Keywords:** hb, esr, crp, alp, rcc, bone metastasis, skeletal-related events

## Abstract

Introduction

Skeletal metastasis is catastrophic in patients with renal cell carcinoma (RCC), leading to skeletal-related events (SRE) such as nerve entrapment, hypercalcemia and even pathological fractures, which may require surgical intervention. The nature of the bone metastasis in advanced RCC is large, destructive, hyper-vascular and mostly lytic. The present retrospective analysis aims to identify potential risk factors for predicting SREs in advanced RCC with bone metastasis.

Methods

The clinical data of 42 patients with RCC and bone metastasis and at least one episode of SRE were reviewed, and the correlations between erythrocyte sedimentation rate (ESR), alkaline phosphatase (ALP), C-reactive protein (CRP), haemoglobin (Hb), carcinoembryonic antigen (CEA) and bone metastases were analysed. Risk factors were identified by multivariate logistic regression analysis. Bone metastasis was diagnosed on a bone scan. The receiver operating characteristic (ROC) curve calculated the cut-off value of the independent correlation factors.

Results

The areas under the ROC curve for ALP, Hb, CRP, and ESR were 0.84, 0.76, 0.86 and 0.88, respectively, suggesting excellent discriminatory capability of ALP, CRP, ESR and sufficient discriminative ability of Hb in predicting bone metastasis. Multivariate logistic regression analysis showed ALP, CRP, Hb and ESR associated with SRE and skeletal metastasis.

Conclusion

We propose that an A.C.H.E. score encompassing ALP, CRP, Hb, and ESR are potential risk factors for developing SRE and concomitant bone metastasis in advanced RCC patients. For new RCC patients, if values of ALP >128 U/L, CRP ≥74 mg/L, Hb <11.5 g/L, and ESR ≥55 mm/hr are detected, intensive monitoring and bone scanning are warranted as these cases are at a higher risk of skeletal events.

## Introduction

Renal cell carcinoma (RCC) is often diagnosed behindhand due to non-specific symptomatology. One in five newly diagnosed cases has distant metastases. Nearly 70% of RCC patients are eventually associated with bone metastasis. RCC is the sixth most commonly found cancer in males and the 10th most frequently diagnosed cancer in women and accounts for 2-3% of all cancers overall. Of all the cases of RCC diagnosed, one-third of them present with metastasis, of which one-third present with bone metastasis. The most frequent locations for bone metastasis in RCC are the vertebral column, sacrum, pelvis, and proximal femur. In the spinal region, the lumbar segment is most frequently involved, after the thoracic and cervical segments [1].

Skeletal-related events (SREs) are defined as pathological fractures, surgical intervention, the requirement for palliative radiotherapy to the bone, spinal cord compression or hypercalcemia [2]. In RCC, bone is the second most affected site by metastasis, only after lung metastasis. RCC is very aggressive and destructive, resulting in more skeletal-related events than bone metastasis compared to other urothelial cancers [3]. Bone metastasis in RCC is often osteolytic and causes skeletal-related events like pain, pathological fractures, spinal cord compression and hypercalcemia. It is well acknowledged that some treatments, such as radiation therapy, can cause sclerotic alterations in osteolytic bone lesions. Five-year and 10-year survival rates in metastatic RCC are less than 10% and 5%, respectively, whereas the five-year survival rate in solitary bone metastasis from RCC is reportedly between 35% and 60% [4,5]. Hence early diagnosis and timely intervention in patients with bone metastasis offer a survival advantage.

Currently, the diagnosis of bone metastasis is based on bone scan, contrast-enhanced computed tomography (CECT) and MRI, which is costly, and early bone metastatic lesions might not be easily detected through these techniques. Hence several biochemical parameters, pro-inflammatory tumour markers and clinical-pathological factors have been studied to prognosticate SRE’s bone metastasis. Though some correlation has been ascertained with factors like alkaline phosphatase (ALP) [6], serum calcium [7], haemoglobin (Hb) [8] and bone metastasis, no definite scoring system with well-defined cut-off values for various parameters has been proposed. Hence this study aims to identify the potential risk factors for predicting SREs in advanced RCC with bone metastasis in newly diagnosed patients and those who have already received treatment and to establish the correlation between diverse clinical factors and aggressiveness of bone metastasis secondary from metastatic RCC.

## Materials and methods

The study was conducted in accordance with the Declaration of Helsinki, and approved by the Institutional Review Boards (or Ethics Committee) of Gauhati Medical College Hospital, Guwahati, Assam, India (MC/232/2016/34). Informed consent was obtained from all subjects involved in the study.

We performed a retrospective analysis on 42 metastatic RCC patients who received treatment at the Department of Urology between January 2010 and December 2016. We selected the patients in this study based on 1) Patients who were diagnosed histologically for RCC with methylene diphosphonate technetium-99 (MDP TC 99) bone scan demonstrating at least one site of metastasis to bone and at least one episode of SRE within five years of follow-up; 2) Availability of data on complete blood biochemical measurements including information on C-reactive protein (CRP), cancer antigen (CA) 15-3, lactate dehydrogenase (LDH), erythrocyte sedimentation rate (ESR), alpha-fetoprotein (AFP), ALP, carcinoembryonic antigen (CEA), CA125, and CA19-9 done within one week before planning definitive management. Patients without adequate blood reports before surgical resection, patients with inadequate follow-up, and patients with active inflammatory disease were not included in the study. Skeletal-related events due to bone metabolic disorders, hyperparathyroidism, and hepatic dysfunction were excluded from the study. Using a common data extraction method, the baseline clinical, pathologic, and biochemical information were gathered, including age at the time of surgery, gender, tumour size, tumour stage (T stage), tumour necrosis (TN), and Fuhrman's grade (FG). Tumour grade was evaluated using the Fuhrman grading system, and the T stage was established using the 2010 TNM classification of malignant tumour staging system. All patients were risk-stratified according to the Memorial Sloan Kettering Cancer Center (MSKCC) grading system.

The correlation between clinic-pathological variables and tumour markers mentioned above was studied in patients of RCC with bone metastasis and was statistically analyzed by a multivariate logistic regression model. The Chi-square test assessed qualitative variables. The correlation between variables was evaluated using Pearson's correlation coefficient. The sensitivity and specificity were computed based on optimal cut-off scores from the area under the curve (AUC), calculated from receiver operating characteristic (ROC) curves. To find independent risk factors for predicting SREs in RCC patients with bone metastases, a multivariate logistic regression analysis was performed using variables having a p-value of less than 0.05 in the univariate analysis. Data having a p-value less than 0.05 were considered statistically significant. All analysis was performed by IBM SPSS Version 21.0 (IBM Corp., Armonk, NY, USA).

## Results

Forty-two cases of metastatic RCC were included in the retrospective study with a special emphasis on metastatic skeletal sites. Thirteen patients (30.95%) had upfront bone metastasis in our analysis of the total study population. The mean follow-up in the study was 42±9.5 months. The entire study population was distributed in two groups with two or fewer bony metastases and more than two bony lesions. The male/female ratio in the study was 5 to 1, with 35 males and seven females, with the mean age for men being 57.12±4.15 years and the mean age for women being 54.17±2.5 years (p=.008) (Table 1). Right- and left-side renal system was involved in 54.76% (n=23) and 45.24% (n=19) cases respectively (Table 1). Metastasis to lung and lymph node were seen in 52.3% (n=22) and 50% (n=21) respectively alongside bone metastasis. Histopathology report showed clear cell carcinoma in 30 (71.3%) patients and sarcomatoid differentiation in 22 patients (52.3%). Spine (n=22, 52.3%) was the most common site of skeletal metastasis followed by ribs (n=6, 14.2%), femur (n=6, 14.2%), pelvis (n=4, 9.5%) and sternum (n=4, 9.5%). Skeletal metastasis with two or fewer lesions was seen in 20 (47.6%) patients while multiple skeletal metastases (more than two lesions) were seen in 22 (52.3%) patients. MSKCC's poor risk category had 30 patients, while the intermediate risk category had 12 patients. The mixed pattern of bone metastasis patients was present in the study with lytic bone metastasis being discovered in the CECT scan and blastic ones being detected in the Tc 99 mdp bone scan. 

**Table 1:** Demographic and clinical profile of advanced RCC patients with bone metastasis with simultaneous SRE Beta (b) coefficient values and absolute numbers (n) for identifying significant demographic and clinical factors implicated in patients with bone metastasis and simultaneous SRE using binary logistics regression analysis test. BM: bone metastases, RCC: renal cell carcinoma, SRE: skeletal-related events

Parameter	Number of Bone Metastases		X2	p-value
	≤ 2 BM beta coefficient value, (n)	>2 BM beta coefficient value, (n)		
Age (years)				
<60	13218 (8)	250 (8)	1.313	0.21
>60	10216 (14)	177 (12)		
Sex				
Female	8771 (4)	121 (3)	6.712	0.008
Male	17121 (18)	291 (17)		
Grade				
I -II	15919 (2)	115 (7)	181.19	<0.001
III-IV	8057 (10)	338 (23)		
T Stage				
T1-T2	21515 (4)	175 (6)	236	<0.002
T3-T4	6016 (13)	255 (19)		
Laterality				
Left	13123 (10)	255 (9)	2.424	0.187
Right	14220 (12)	239 (11)		
Histology				
Clear cell (CC)	21222 (16)	519 (14)	111.214	<0.003
Chromophobe (CP)	1311 (2)	7 (2)		
Papillary (PL)	42 (2)	6 (3)		
Collecting Duct (CD)	37 (2)	21 (1)		
Tumour Size				
T1a	11918 (2)	41 (3)	325	<0.001
T1b	9212 (3)	188 (2)		
>T2	4912 (17)	311 (15)		

The male population was more dispensable towards early bone metastasis as compared with the female population when the period was calculated from the first radiological confirmation [43 months (male) versus 35 months (female), p=0.373]. The comparable histological features between the two groups are mentioned in Table 1. High Fuhrman grade (III & IV, n=33) and a higher T stage (>T2, n=32) were significantly associated and correlated with the increased number of bony lesions in the study population (p<.001). Skeletal girdle pain was a normal finding in both the study groups (p=.03), which responded partially to oral opioid analgesics. The character of the pain was somatic (achy, sharp, well-localized) in 10 patients. At the same time, it was neuropathic (i.e., burning, shooting, radiating) in 12 cases, while in the remaining 20 patients, it was a mixed picture between the two extremes (p=0.142).

**Figure 1:**
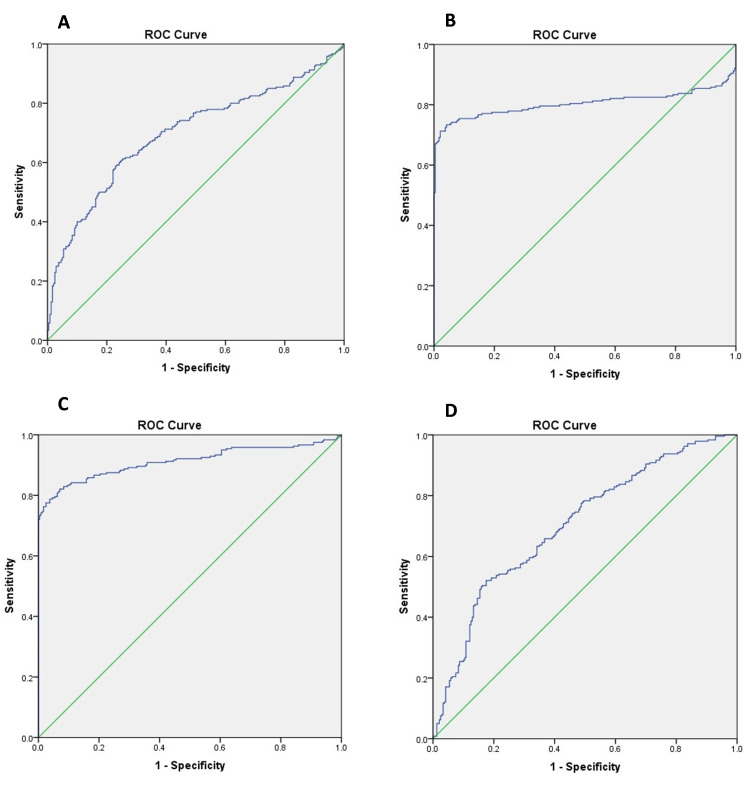
ROC curve for ALP (A), CRP (B), ESR (C) and Hb (D) to measure the cut-off level for the determination of bone metastasis in advanced RCC patients with simultaneous SRE ROC: receiver operating characteristic, ALP: alkaline phosphatase, CRP: C-reactive protein, ESR: erythrocyte sedimentation rate, Hb: haemoglobin, RCC: renal cell carcinoma, SRE: skeletal-related events

The Chi-square test for dichotomous data and the student’s t-test for continuous data were used to identify the potential risk factors for bone metastases. The data were analysed through the ROC curve to determine the accuracy, sensitivities and specificities of predicting bone metastases by these risk factors. The cut-off values of ALP, CRP, Hb and ESR for the prediction of bone metastasis were >236 U/ml (AUC .849, p=.01), >84 µg/ml (AUC .856, p=.021), <10.5 g/l (AUC .765, p=.045) and 74 mm/hour (AUC .880, p=.032), respectively (Figure 1). 

The results showed that there was a significant difference between the groups varying on bone metastatic sites of less than or equal to two and more than two (Table 2). ALP, CRP, and ESR serum concentrations were significantly higher in patients who had experienced SREs with bone metastases than those without bone metastases (P). Nonetheless, the serum level of Hb in more than two bone metastatic sites was statistically lower than those of non-bone metastases (Table 2). The mean ± SD for various biochemical risk factors differentiating between the two risk groups is given in Table 2. The sensitivities and specificities of the combination of risk factors for predicting SRE were also calculated, and a variety of four elements was the most accurate in predicting SRE. 

**Table 2:** Group characteristics based on the number of bone metastasis sites with serum pro-inflammatory markers BM: bone metastases, CEA: carcinoembryonic antigen, ALP: alkaline phosphatase, CRP: C-reactive protein, AFP: alpha-fetoprotein, CA: cancer antigen, Hb: haemoglobin, ESR: erythrocyte sedimentation rate

Parameter	Number of Bone Metastases		t/X^2 ^value	p-value
	≤ 2 BM	>2 BM		
CEA	3.00 ±3.54	34.18 ±1.13	0.990	0.342
ALP	248±140	327 ± 189.3	6.34	0.01
CRP	98 ± 3.25	114 ±7.25	5.06	0.02
AFP	3.47±6.07	2.86 ± 1.17	0.225	0.823
CA-125	62.10 ± 236.65	31.09 ± 25.40	0.451	0.653
CA-153	16.50±18.24	15 ± 7.97	0.251	0.803
Hb	10.3 ± 1.40	9.8 ±1.25	7.16	0.01
ESR	78 ± 4.25	94 ±6.50	4.56	0.03

Based on the analysis, the concentration of ESR had the highest predictive accuracy for more than two bone metastases among these factors (AUC=0.88), with a sensitivity of 87.62% and specificity of 88.97%. Nonetheless, as a single risk factor, Hb had low accuracy in predicting bone metastases (AUC=0.76) compared with the other three significant factors (ALP, ESR, CRP) with a sensitivity of 55.9% and specificity of 79.4%. The mean ± SD for ALP for diagnosing more than two bone lesions was 327±189.3 u/ml (p=.01), while the same for CRP was 114±7.25u/ml (p=.02). The mean ± SD (Hb) for diagnosing more than two bone lesions was <9.8±1.25 (p=0.01), while the same for ESR was 94±6.50 AEFH (p=.03).Additionally, multivariate logistic regression analysis indicated that in more than two bony metastatic sites ALP, CRP, ESR and Hb were the independent risk factors for predicting skeletal-related events in patients with bone metastases with RCC. On the multivariate logistic regression analysis calculated to determine bone metastasis along with SREs, the odds ratio (OR) for serum ALP (95% CI) was 2.48 (1.49-4.02), p=.001, while the same for CRP (95% CI) was 2.28 (1.08-4.81), p=.029. The OR for Hb (95% CI) was .302 (.125-.729), p=.008, while the same for ESR (95% CI) was 2.32 (1.32-3.55), p=.042 (Table 3). 

**Table 3:** Comparison of groups based on multivariate logistic regression analysis to determine causative agents for prediction of SRE’s in advanced RCC with bone metastasis ALP: alkaline phosphatase, CRP: C-reactive protein, Hb: haemoglobin, ESR: erythrocyte sedimentation rate, SRE: skeletal-related events, RCC: renal cell carcinoma, OR: odds ratio, CI: confidence interval

Parameters	β	OR	OR (95% CI)	X^2^	p-value
ALP	0.895	2.488	1.491-4.02 1	12.52	< 0.001
CRP	0.828	2.289	1.088-4.817	4.76	0.029
Hb	— 1.1 96	0.302	0.125—0.729	7. 10	0.008
ESR	0.7902	2.323	1.322-3.554	6.56	0.042

## Discussion

There is an increased rate of development of SREs in advanced RCC compared to other genitourinary cancers, and it requires an increased awareness of these events in tertiary care centres. The data included in our background research explores the possibility of statistically concluding the rate of development of SREs in patients with metastatic RCC to the bone. Many biochemical triggers and immunological escape mechanisms are implicated in developing SREs in patients with metastatic RCC [9]. Multiple pro-inflammatory markers increase the potential for tumour cells to preferentially metastasise to bone, including the changes induced by the tumour cells or their products in the bone microenvironment [10]. Based on the radiologic appearance of predominant bone destruction or deposition of new bone, bone metastases are commonly grouped as osteolytic or osteoblastic. Many patients have a mixed picture of osteolytic and osteoblastic metastases and are characterised by dysregulation of the normal bone remodelling process. Published literature reports suggest that metastatic disease mainly involves areas of red bone marrow, such as the axial skeleton, skull or the medullary portion of the appendicular skeleton [11,12]. The process of bone metastasis development begins with colonisation when circulating tumour cells, along with the help of inflammatory biomarkers such as CRP and ALP, enter the bone marrow compartment and engage in specialised microenvironments or niches [13]. Further, the tumour cells evade the immune response and escape from the dormant state to actively proliferate and form micro-metastases by re-stimulation, which again involves the help of pro-inflammatory biomarkers. At last, cells grow uncontrollably, turn independent of the tumour niche, and ultimately cause bone modifications as the metastasis expands. 

Positron emission tomography-computed tomography (PET-CT) and whole-body MRI are time-consuming, costly for patients, and limited by low specificity and sensitivity. Technetium 99 MDP bone scan is an accurate method for detecting distant bone metastases in patients with RCC; nonetheless, it is affected by the flare phenomenon [14]. Inflammatory serum tumour markers examinations have the advantage of being repeatable and low-cost for patients. Therefore, we retrospectively analysed the biochemical serum pro-inflammatory biomarkers in patients with bone metastasis and simultaneous SREs in metastatic RCC.

In the present study, 42 patients with advanced RCC who had at least one bone metastatic site and one episode of SRE were retrospectively analysed. The present study reported that patients with more than two bone metastasis had a high incidence of FG 3 and FG 4 (n=33, 78.57%) along with high T stage (>T2) (n=32, 76.19%, Table 1). Our study’s clear cell histology variant was 30 patients (71.43%). The share of sarcomatoid differentiation in our present study was 52.38% (n=22, p=.06). A previous study screened the records of more than 1800 patients who died from RCC, finding 398 patients (22%) with bone metastasis and showed that the majority of patients with bone metastasis at the time of RCC diagnosis had high Fuhrman grading and a higher T stage [15-17]. A recent study by Stellato and colleagues also found that patients with high Fuhrman grade and high T stage at the time of diagnosis had the highest incidences of bone metastases in RCC. Additional analysis showed that the clear cell histology variant (n=20) was significantly associated with an increased incidence of bone metastasis (Chi-square coefficient - 111.21, p=<.003) and was one of the risk factors for SREs in advanced RCC patients [18]. A previously reported study by Santoni et al. [19] also found that patients with aggressive clear cell RCC at the time of presentation had the highest incidences of bone metastases and subsequent SREs requiring intervention in advanced RCC. The authors in the present analysis propose that a high Fuhrman’s grade, >T2 stage and clear cell histology are reliable factors for predicting SREs accurately in advanced RCC.

The incidence of bone metastases from RCC at the primary diagnosis in our study was 30.85% (n=13), which was a little lower than that reported in Chen et al. study at 35%, which was based on significant population analysis [19]. In addition, we found that most of the patients with advanced RCC were 50-69 years (Table 1) which was confirmed in a previous analysis by Wang and colleagues [20]. A previous study documented that the intensity of the pain may vary depending on the presence or absence of neuromas as part of tumour-induced bone remodelling, endosteal nerve compression by tumour, nerve injury from the extension of the bone metastasis out of the bone, and location of the metastasis within the bone [21]. In our analysis, sudden severe pain was caused by documented pathologic fracture in seven cases, while neurologic symptoms were present in patients with vertebral metastases (n=22). The proposed mechanism reported vertebral metastasis causing spinal cord compression and symptoms ranging from pain to neurologic deficits, including motor weakness and paralysis, sensory deficits, bowel and bladder dysfunction, and ataxia [22]. Previous literature also reported that patients with advanced RCC had a significant risk of developing SRE with increasing age and multiple bone metastasis sites. The metastatic skeletal distribution in our study was in coherence with previously reported literature [23].

In addition to the histological parameters, we identified that the serum levels of ALP, CRP, Hb and ESR are directly associated with bone metastasis. These were the risk factors for detecting SREs in patients with advanced clear cell RCC. A past study has reported that higher histopathological characteristics when involved with high serum pro-inflammatory biomarkers had more propensity towards early and multiple bone metastasis and increased SRE (OR, 95% CI), 2.52 (0.80-4.25)[24]. ALP is an intrinsic bone formation pro-inflammatory marker, which is the most used for detecting increased bone turnover in metastatic breast and prostate cancer. ALP is well known as a tumour marker for ovarian cancer but rarely evaluated in the early detection of bone metastases in advanced RCC. In another recent study among cases of gastric adenocarcinoma with bone metastasis, bone ALP was significantly higher in the patient group (57.32±46.83 vs 34.57±21.57, p=0.037) than in the control group. Bone ALP was positively associated with ALP, osteocalcin and negatively associated with 25(OH) D. According to ROC-curve analysis, at the threshold value 29.60 μg/L, the sensitivity of bone ALP was 76.7%, and the specificity was 59.4% [25]. In the current study, the serum concentration of ALP was identified as a marker for bone metastases and a risk factor for SRE in advanced RCC, with a cut oﬀ value >236 U/l, with sensitivity and speciﬁcity of 87.9% and 73.5% respectively (p=.01) in the ROC analysis. The sensitivity and specificity of our ROC analysis matched the study, as mentioned earlier.

CRP plays a vital role in the inflammatory process by binding to Fc receptors to induce the release and elevated activity of pro-inflammatory cytokines such as interleukin (IL)-6 [26]. Pro-inflammatory cells are tumour promoters, produce a congenial environment for tumorigenesis/angiogenesis, and favour neoplastic spread and metastasis. CRP causes an increased level of inflammatory cytokines, such as IL-1, IL-6, and tumor necrosis factor-alpha (TNF-α) and induces an imbalance of the RANK/RANKL/OPG system, known for regulating the differentiation of osteoclasts and osteoblasts to reduce the total BMD [27]. These results indicated that CRP could indicate bone turnover and be a possible marker of bone metastasis. In the present analysis, the concentration of CRP in patients with bone metastases was significantly higher than in those without bone metastases. Our study indicated that serum CRP was an independent risk factor for bone metastasis and SRE in patients with advanced RCC at cut-oﬀ value >84 U/L (p=.021), and the sensitivity and speciﬁcity was 75.9% and 85.5% respectively at ROC analysis. 

ESR is significantly elevated in malignant tumours, especially in aggressive and rapidly growing tumours, and represents an independent risk factor for many tumours [27]. Previous analysis has reported that ESR >20 mm/AEFH can be an independent risk factor for distinguishing invasive and non-invasive prostatic adenocarcinoma [28]. In the study mentioned above, the level of ESR in the bone metastasis group was significantly higher than in the non-bone metastasis group with ESR >23.5 mm/h, having the maximum probability of bone metastasis with a sensitivity of 77.3%, and the specificity was 75.6%. The results of our study indicated that abnormally high ESR levels significantly increased the risk of bone metastases and SREs in advanced RCC. The cut-off value was >74 mm/AEFH (p=.032) with sensitivity and speciﬁcity of 63.9% and 76.9% respectively.

Past studies have reported that low Hb commonly affects more than 50% of malignancy patients. A past study found a component in bone related explicitly to the metastasis of adenocarcinoma prostate to the bone and found as haemoglobin [29]. Another previous study reported that Hb concentration was of prognostic importance for patients with early breast cancer [30]. In our study, the Hb level was significantly lower in advanced RCC with bone metastasis compared to those without bone metastases, and it was identified as one of the risk factors for SREs. The rationale behind bone metastases and low Hb would be bone marrow dysfunction, which decreases hematopoietic function. In previous studies, the predictive accuracy for bone metastases by haemoglobin was low (AUC=0.316), but in our analysis, the cut oﬀ value was <10.5 g/L with sensitivity and speciﬁcity being 55.9% and 79.4% respectively.

**Figure 2:**
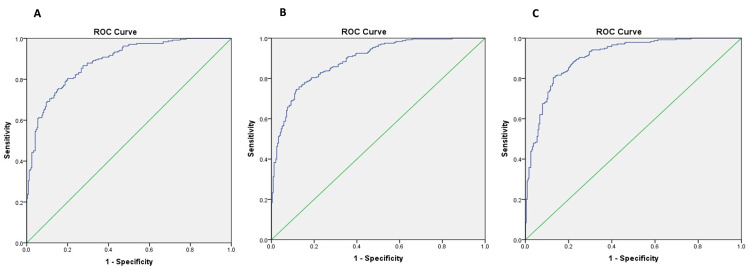
Depicting ROC curve analysis for the combination of risk factors: (A) for a combination of Hb and ESR; (B) for a combination of ALP, CRP, and ESR; and (C) for a combination of ALP, CRP, Hb and ESR. ROC: receiver operating characteristic, Hb: haemoglobin, ESR: erythrocyte sedimentation rate, ALP: alkaline phosphatase, CRP: C-reactive protein

Besides evaluating the single pro-inflammatory serological risk factor for predicting SRE in advanced RCC, we also assessed the predictive accuracy of combining risk factors in this study. Based on the current analysis, combined ALP, CRP, and Hb with ESR were the most accurate for predicting bone metastases and SREs. To investigate the predictive accuracy of combining risk factors for SREs in patients with advanced RCC with bone metastasis, ROC analysis was conducted for a different combination of risk factors (Figure 2A-2C). For combined ALP, CRP, Hb and ESR, the prediction of SREs was more accurate than other combinations (AUC=0.900). Among the three combinations of risk factors, combined ALP, CRP, and ESR were second in line for predicting the same bone (AUC=0.892). Additionally, haemoglobin and ESR had the most negligible predictive value among the three combinations of risk factors (AUC=0.882) (Table 4). When the study population was distributed in MSKCC risk protocol, none of the cases was in the favourable risk group. In contrast, 12 cases landed in the intermediate risk group, while 30 were allotted to the poor risk category. The combination of risk factors such as ALP, CRP and ESR were significantly associated (p=.003) but not correlated (Pearson R=.0588, p=.09) with more than two bone metastatic sites and development of SRE in the intermediate risk group but was significantly associated (p=.001) and correlated with the high-risk group (Pearson R=.425, p=.002) as well. 

**Table 4:** AUC (area under the curve) analysis for combination of serum pro-inflammatory markers for prediction of SREs in advanced RCC with bone metastasis. Hb: haemoglobin, ESR: erythrocyte sedimentation rate, ALP: alkaline phosphatase, CRP: C-reactive protein, SRE: skeletal-related events, RCC: renal cell carcinoma

Parameters Combined	AUC	Sensitivity	Specificity	p-value
Hb + ESR	0.882	81.7	82.4	< 0.001
ALP + CRP + ESR	0.892	76	88.9	0.029
ALP+CRP+Hb+ESR	0.900	78.7	87.8	0.008

The combination of risk factors such as ALP, CRP, Hb and ESR were significantly associated (p=.001) and correlated (Pearson R=0.345, p=0.008) with more than two bone metastatic sites and development of SRE in the intermediate risk group and was significantly associated (p=0.002) and correlated with the high-risk group (Pearson R=0.488, p=0.001) as well. 

## Conclusions

In addition to scoring the single risk factor for predicting SREs in advanced RCC with bone metastases, we also evaluated the predictive accuracy of combining the risk factors in this study. Based on our analysis, combined serum ALP, CRP, Hb and ESR were the most accurate for predicting the same. The authors conclude that the most frequent site of bone metastasis in advanced RCC patients is the spine. All new and existing cases with ALP >236 U/L, CRP >84 µg/mL, Hb <10.5 g/L, and ESR >74 mm/AEFH require intensive monitoring for the possible occurrence of bone metastasis and concomitant SREs. The combination (A.C.H.E.) has the highest sensitivity and specificity for predicting bone metastasis and SREs. Understanding these mechanisms has revealed new potentially beneficial diagnostic approaches to predict bone metastases in various malignancies, especially RCC.

A few constraints were also present in the study. First and foremost, this study was based on a single-institutional database, although inclusion criteria were formulated to decrease the selection bias. Secondly, it was a retrospective analysis, and some data attrition was unavoidable, which may have affected the analysis results. Some data, such as patients’ progression-free survival rate and overall survival rate, were not included in this study; therefore, a prospective, multicentre cohort study is helpful to validate the results of our research.

## References

[1] Murai M, Oya M (2004). Renal cell carcinoma: etiology, incidence and epidemiology. Curr Opin Urol.

[2] Patard JJ, Leray E, Rodriguez A, Rioux-Leclercq N, Guillé F, Lobel B (2003). Correlation between symptom graduation, tumor characteristics and survival in renal cell carcinoma. Eur Urol.

[3] Miyao N, Naito S, Ozono S (2011). Late recurrence of renal cell carcinoma: retrospective and collaborative study of the Japanese Society of Renal Cancer. Urology.

[4] Beuselinck B, Oudard S, Rixe O (2011). Negative impact of bone metastasis on outcome in clear-cell renal cell carcinoma treated with sunitinib. Ann Oncol.

[5] Santoni M, Conti A, Procopio G (2015). Bone metastases in patients with metastatic renal cell carcinoma: are they always associated with poor prognosis?. J Exp Clin Cancer Res.

[6] Kume H, Kakutani S, Yamada Y (2011). Prognostic factors for renal cell carcinoma with bone metastasis: who are the long-term survivors?. J Urol.

[7] Hofbauer LC, Khosla S, Dunstan CR, Lacey DL, Boyle WJ, Riggs BL (2000). The roles of osteoprotegerin and osteoprotegerin ligand in the paracrine regulation of bone resorption. J Bone Miner Res.

[8] Chen XY, Lan M, Zhou Y (2017). Risk factors for bone metastasis from renal cell cancer. J Bone Oncol.

[9] Ito H, Shioi K, Murakami T (2012). C-reactive protein in patients with advanced metastatic renal cell carcinoma: usefulness in identifying patients most likely to benefit from initial nephrectomy. BMC Cancer.

[10] D'Oronzo S, Coleman R, Brown J, Silvestris F (2019). Metastatic bone disease: pathogenesis and therapeutic options: up-date on bone metastasis management. J Bone Oncol.

[11] Mikami S, Katsube K, Oya M (2009). Increased RANKL expression is related to tumour migration and metastasis of renal cell carcinomas. J Pathol.

[12] Mukai S, Yorita K, Kawagoe Y (2015). Matriptase and MET are prominently expressed at the site of bone metastasis in renal cell carcinoma: immunohistochemical analysis. Hum Cell.

[13] Kominsky SL, Doucet M, Brady K, Weber KL (2007). TGF-beta promotes the establishment of renal cell carcinoma bone metastasis. J Bone Miner Res.

[14] Weber K, Doucet M, Kominsky S (2007). Renal cell carcinoma bone metastasis--elucidating the molecular targets. Cancer Metastasis Rev.

[15] Joeckel E, Haber T, Prawitt D (2014). High calcium concentration in bones promotes bone metastasis in renal cell carcinomas expressing calcium-sensing receptor. Mol Cancer.

[16] Xie C, Li Y, Li Q (2015). Increased insulin mRNA binding protein-3 expression correlates with vascular enhancement of renal cell carcinoma by intravenous contrast-CT and is associated with bone metastasis. J Bone Oncol.

[17] Satcher RL, Pan T, Cheng CJ (2014). Cadherin-11 in renal cell carcinoma bone metastasis. PLoS One.

[18] Stellato M, Santini D (2021). Metastatic renal cell carcinoma classified as good risk: the easiest choice has become the hardest. Chemotherapy.

[19] Santoni M, Berardi R, Amantini C, Burattini L, Santini D, Santoni G, Cascinu S (2014). Role of natural and adaptive immunity in renal cell carcinoma response to VEGFR-TKIs and mTOR inhibitor. Int J Cancer.

[20] Wang J, Zhao X, Qi J, Yang C, Cheng H, Ren Y, Huang L (2015). Eight proteins play critical roles in RCC with bone metastasis via mitochondrial dysfunction. Clin Exp Metastasis.

[21] Chen SC, Kuo PL (2016). Bone metastasis from renal cell carcinoma. Int J Mol Sci.

[22] Haber T, Jöckel E, Roos FC (2015). Bone metastasis in renal cell carcinoma is preprogrammed in the primary tumor and caused by AKT and Integrin α5 signaling. J Urol.

[23] Wotschofsky Z, Liep J, Meyer HA (2012). Identification of metastamirs as metastasis-associated microRNAs in clear cell renal cell carcinomas. Int J Biol Sci.

[24] Kohno N, Aogi K, Minami H (2005). Zoledronic acid significantly reduces skeletal complications compared with placebo in Japanese women with bone metastases from breast cancer: a randomized, placebo-controlled trial. J Clin Oncol.

[25] Costa L, Major PP (2009). Effect of bisphosphonates on pain and quality of life in patients with bone metastases. Nat Clin Pract Oncol.

[26] Ullen A, Schwarz S, Lennartsson L (2009). Zoledronic acid induces caspase-dependent apoptosis in renal cancer cell lines. Scand J Urol Nephrol.

[27] Alcaraz A, González-López R, Morote J (2013). Biochemical markers of bone turnover and clinical outcome in patients with renal cell and bladder carcinoma with bone metastases following treatment with zoledronic acid: the TUGAMO study. Br J Cancer.

[28] Dhillon S, Lyseng-Williamson KA (2008). Zoledronic acid : a review of its use in the management of bone metastases of malignancy. Drugs.

[29] Rosen LS, Gordon D, Kaminski M (2001). Zoledronic acid versus pamidronate in the treatment of skeletal metastases in patients with breast cancer or osteolytic lesions of multiple myeloma: a phase III, double-blind, comparative trial. Cancer J.

[30] Broom RJ, Hinder V, Sharples K (2015). Everolimus and zoledronic acid in patients with renal cell carcinoma with bone metastases: a randomized first-line phase II trial. Clin Genitourin Cancer.

